# Hyperuricemia Associated with Thiazide Diuretics in Hypertensive Adults

**DOI:** 10.7759/cureus.5457

**Published:** 2019-08-22

**Authors:** Ravi Raja, FNU Kavita, FNU Amreek, Ali Shah, Khalid A Sayeed, Alina Sehar

**Affiliations:** 1 Internal Medicine, New Medical Center, Al Ain, ARE; 2 Medicine, Liaquat University of Medical and Health Sciences, Jamshoro, PAK; 3 General Surgery, New York University Langone Medical Center, New York, USA; 4 Internal Medicine, Jinnah Sindh Medical University, Karachi, PAK; 5 Medicine, Liaquat College of Medicine and Dentistry, Darul Sehat Hospital, Karachi, PAK; 6 Internal Medicine, United Medical and Dental College, Karachi, PAK

**Keywords:** pakistan, hypertension, thiazide, serum uric acid levels, hyperuricemia, anti-hypertensive drugs, diuretics

## Abstract

Introduction

Thiazide diuretics are essential first-line anti-hypertensive drugs which not only maintain blood pressure but also reduce stroke and congestive heart failure associated with morbidity and mortality in hypertensive patients. However, thiazide diuretics are associated with elevated serum uric acid (SUA) levels. This study aimed to evaluate the impact of thiazide diuretic use on their SUA levels among hypertensive individuals of Pakistan.

Methods

In this cross-sectional, prospective study, adult hypertensive patients were recruited. They were divided into two groups - thiazide diuretic group and non-thiazide group. Demographic characteristics, hypertension-related characteristics, and SUA levels were included. Data were then entered and analysed using SPSS for Windows version 22.0 (IBM Corp., Armonk, NY, USA).

Results

In the thiazide group, 24.5% were hyperuricemic as compared to 15.3% in the non-thiazide group (p=0.03). The overall mean SUA levels in the thiazide group were significantly higher than those in the non-thiazide group (5.9 ± 2.1 vs. 5.3 ± 2.7 mg/dL; p=0.02). Males in the thiazide group also showed a similar pattern (5.9 ± 2.3 vs. 5.1 ± 2.1 mg/dL; p=0.02); however, the differences were insignificant in females. Patients using thiazide diuretics for one to three years were more non-hyperuricemic than hyperuricemic (p=0.000). Among hyperuricemic patients, 36.5% were taking thiazides for three to four years and 46% were taking them for more than four years (p<0.05).

Conclusion

Hyperuricemia is a more common occurrence in thiazide diuretic users as compared to non-users. The overall sample, and men using thiazide diuretics, reported a higher mean SUA as compared to non-users. As the years of thiazide usage advanced, the number of hyperuricemic participants also significantly increased.

## Introduction

In almost all patients with hypertension (HTN), renal sodium retention is the primary pathology contributing to elevated blood pressures (BP). Thiazide diuretics are essential first-line anti-hypertensive drugs as they help increase urinary sodium excretion [1]. Diuretic agents are essential in reducing morbidity and mortality related to stroke and congestive heart failure in patients with HTN. However, despite patients achieving adequate control with diuretics, these drugs are also associated with adverse events [2]. Thiazide diuretics are associated with elevated serum uric acid (SUA) levels. They increase direct urate reabsorption in the proximal renal tubules [3]. Elevated SUA is an independent risk factor for gout [2].

These agents increase the levels of SUA and thus may contribute to the risk of gout. While HTN is the main indication of thiazide diuretic use, it is also an independent risk factor for gout [4]. In contrast, hyperuricemia is an independent risk factor for HTN [5]. Many observational studies have been conducted to study their correlation. However, studies were unable to differentiate whether it was the diuretic use which caused gout or HTN leading to bias [6]. Some studies did not establish the type of diuretic leading to gout [4]. In a Pakistani observational study, both HTN and use of thiazide diuretics were established as independent risk factors for hyperuricemia in this population [7]. However, to the best of our knowledge, there has been no study conducted among hypertensive individuals of Pakistan to evaluate the impact of thiazide diuretic use on their SUA levels. This will not only add to very limited local data available but also assist physicians in weighing risk of hyperuricemia before prescribing thiazide.

## Materials and methods

A cross-sectional, prospective study was conducted in the outpatient department (OPD) of a public hospital in Pakistan. The study was approved by the ethics review committee, and informed consent was taken from all participants. The study duration was from July to December 2018.

In this study, patients of both genders and age ≥18 years visiting the OPD for routine follow-up of HTN were invited to participate. Patients who were diagnosed with HTN and were taking anti-hypertensive drugs for at least one year were included. Patients were randomized into two groups. One group comprised of participants taking any angiotensin-converting enzyme inhibitor (ACEi) or angiotensin receptor blocker (ARB) along with a thiazide diuretic and were named the “thiazide group.” All patients included in the thiazide group were taking their thiazide diuretic for at least one continuous year. The non-thiazide group included participants taking ACEi or ARB alone. Patients taking losartan and calcium channel blockers were excluded as these drugs lower the risk of gout incidence. Patients taking fenofibrate and statins were also excluded as these are uricosuric agents. Patients with a history of gout and/or taking anti-gout drugs were excluded.

Demographic characteristics (age and gender) and HTN-related characteristics of all participants were recorded on a preformed questionnaire. Duration of HTN, duration of thiazide use, and systolic and diastolic blood pressures were noted. SUA levels were measured using UASure Blood Uric Acid Monitoring Kit. Samples with incomplete data were removed. Normal range of SUA level for women was 2.4-6.0 mg/dL and for men it was 3.4-7.0 mg/dL. Data were then entered and analysed using SPSS for Windows version 22.0 (IBM Corp., Armonk, NY, USA). Continuous variables were presented as mean and standard deviation. Categorical variables were presented as frequencies and percentages. Means were compared using the dependent t-test. Frequencies were compared by applying the chi-square test. A p value ≤0.05 was taken as significant.

## Results

The study was completed by 167 participants in the thiazide group and 163 in the non-thiazide group. The thiazide group had more males than females (53.3% vs. 46.7%), and the non-thiazide group had slightly more females (50.3% vs. 59.7%). The non-thiazide group was slightly older and had a longer duration of HTN. A comparison of demographic and HTN-related characteristics between these groups is shown in Table 1.

**Table 1:** Demographic and hypertension-related characteristics of thiazide (n=167) and non-thiazide (n=163) groups Abbreviations: BP: blood pressure; HTN: hypertension; SD: standard deviation

Participant characteristics	Thiazide group (n=167)	Non-thiazide group (n=163)
Gender n (%)		
Male	78 (46.7%)	81 (49.7%)
Female	89 (53.3%)	82 (50.3%)
Age in years n (%)		
Mean	56.2 ± 12.8	53.1 ± 10.2
Less than 40 years	43 (25.7%)	26 (15.9%)
40-60 years	67 (40.1%)	65 (39.8%)
More than 70 years	57 (34.1%)	72 (44.2%)
HTN-related characteristics		
Duration of HTN in years (mean ± SD)	7.4 ± 3. 9	8.1 ± 3.8
Duration of thiazide diuretics usage in years (mean ± SD)	4.5 ± 3.1	Not applicable
Systolic BP in mmHg (mean ± SD)	141.6 ± 23.1	142.7 ± 22.6
Diastolic BP in mmHg (mean ± SD)	89.3 ± 12.2	91.5 ± 17.4

The mean SUA levels were compared as shown in Table 2. The thiazide group showed significantly higher - overall and in males - mean SUA levels as compared to the non-thiazide group (Table 2).

**Table 2:** Mean serum uric levels (mg/dL) in thiazide (n=167) and non-thiazide (n=163) groups

	Thiazide group (n=167)	Non-thiazide group (n=163)	P value
Total	5.9 ± 2.1	5.3 ± 2.7	0.02
Male	5.9 ± 2.3	5.1 ± 2.1	0.02
Female	5.8 ± 2.4	5.3 ± 2.5	0.18

In the thiazide group, 24.5% were hyperuricemic as compared to 15.3% in the non-thiazide group (p=0.03) (Table 3). Males were more frequently hyperuricemic in the thiazide group and females in the non-thiazide group; however, the differences were not statistically significant (Table 3).

**Table 3:** Frequency of hyperuricemia in thiazide (n=167) and non-thiazide (n=163) groups

	Thiazide group (n=167)	Non-thiazide group (n=163)	P value
Total	41/167 (24.5%)	25/163 (15.3%)	0.03
Male	21/78 (26.9%)	14/81 (17.1%)	0.14
Female	20/89 (22.5%)	11/82 (23.5%)	0.12

In both study groups, none of the patients suffered from acute gouty flare. In the thiazide group, 17 (41.4%) of the hyperuricemic participants complained of vague joint pain and stiffness. In the non-thiazide group, 9 (36%) individuals complained so.

The incidence of hyperuricemia was then compared with the duration of thiazide diuretic use in the thiazide group as shown in Table 4. Patients in early years of their thiazide use - one to three years - were more non-hyperuricemic than hyperuricemic (p=0.000). Among hyperuricemic patients, 36.5% were taking thiazides for three to four years and 46% were taking them for more than four years (p<0.05) (Table 4).

**Table 4:** Duration of thiazide diuretic use and incidence of hyperuricemia

Year since using thiazide	Hyperuricemia (n=41; 24.5%)	No hyperuricemia (n=126; 75.4%)	P value
1-2 years (n=28; 16.7%)	4 (9.7%)	24 (19.0%)	0.000
2-3 years (n=48; 28.7%)	3 (7.3%)	45 (35.7%)	0.000
3-4 years (n=39; 23.3%)	15 (36.5%)	24 (19.0%)	0.02
More than 4 years (n=52; 31.1%)	19 (46.3%)	33 (26.2%)	0.01

## Discussion

In this study, individuals were significantly more hyperuricemic and the mean SUA levels were significantly higher in the thiazide group. As the years of thiazide usage advanced, the number of hyperuricemic participants also significantly increased.

To the best of our knowledge, the correlation of thiazide use for HTN with raised SUA levels has not been previously studied in Pakistani individuals. The major strength of this study is that we recruited only hypertensive participants, thus controlling for the confounding factor of HTN, which is also known as a risk factor of hyperuricemia. However, it was an open labelled study which makes its methodology weak. Another important limitation of this study is that it did not monitor drug compliance in case of thiazide users. Dietary habits and alcohol intake were also not controlled in this study, which can impact SUA levels.

In large-scale, population-based studies, a correlation of symptomatic gout has been established in individuals with diuretic use [2,4,8,9]. The Framingham Heart Study, which spanned over 53 years, concluded that diuretic use is an independent risk factor of gout, and it is associated with a 2.4 times relative risk of developing gout in women and a 3.4 times relative risk of developing gout in men [8]. In another population-based study, thiazide diuretic use was associated with a 1.4 times risk of developing gout and a 2.3 times higher risk in patients taking loop diuretics. They also concluded that the longitudinal change in SUA levels was 0.72 mg/dL higher in patients who used diuretics for HTN as compared to those who did not use diuretics (p<0.001) [2]. The Systolic Hypertension in the Elderly Program also concluded that in thiazide-using patients, the three-year increase in the SUA level was 0.90 mg/dL higher than the placebo group [10].

On the other hand, in a case-control study, there was a statistically significant association between the use of diuretics and development of gout which was nullified after adjusting for HTN as a confounding variable [11]. We controlled confounding bias in this study by only recruiting hypertensive patients who were never diagnosed with gout before. Previously, a study was conducted in Pakistan which reported both HTN and the use of thiazide diuretics as independent risk factors of gout. However, this study was conducted in the general population and not particularly hypertensive patients; hence, the confounding bias remains [7].

There have been discrepancies in the results of different observational studies. In a retrospective inception cohort, there were no significant differences in the target SUA levels of gout patients who used diuretics as compared to those who did not use diuretics [12]. However, in another population-based, retrospective, case-control analysis, the odds ratio for incidence of gout in patients taking loop diuretics was 2.6, for thiazide diuretics it was 1.7, for thiazide-like diuretics 2.3, and for combined use of loop diuretics and thiazide diuretics it was 4.6 [13]. Vandell et al. have identified the gene regions associated with a thiazide-induced increase in SUA levels in African American and Caucasian populations [14].

In conclusion, there are strong evidences to support both results - diuretics increase SUA levels and do not have an impact on SUA levels. This sheds lights on the urgent need to design robust prospective, longitudinal case-control trials to establish concrete evidence regarding this correlation. Retrospective analysis with heterogeneous populations and uncontrolled confounding factors should be interpreted with caution by the clinicians.

## Conclusions

Thiazide diuretics are very commonly prescribed to hypertensive patients. HTN itself is an independent risk factor of gout, which in turn is an independent risk factor of cardiovascular and neurovascular diseases. This study has shown the substantial impact of thiazide diuretic use on the SUA levels of patients with HTN. Men on thiazide diuretics reported a higher mean SUA level than non-thiazide users. As the years of thiazide usage advanced, the number of hyperuricemic participants also significantly increased.
